# Impact of COVID-19 lockdown on smoking (waterpipe and cigarette) and participants' BMI across various sociodemographic groups in Arab countries in the Mediterranean Region

**DOI:** 10.18332/tid/155007

**Published:** 2022-11-11

**Authors:** Haleama Al Sabbah, Enas A. Assaf, Zainab Taha, Radwan Qasrawi, Leila Cheikh Ismail, Ayesha S. Al Dhaheri, Maha Hoteit, Ayoub Al-Jawaldeh, Reema Tayyem, Hiba Bawadi, Majid AlKhalaf, Khlood Bookari, Iman Kamel, Somaia Dashti, Sabika Allehdan, Mostafa Waly, Diala Abu Al-Halawa, Rania Mansour, Mohammed Ibrahim, Mariam Al-Mannai

**Affiliations:** 1 Department of Health Sciences, College of Natural and Health Sciences, Zayed University, Dubai, United Arab Emirates; 2 Faculty of Nursing, Applied Science Private University, Amman, Jordan; 3 Department of Health Sciences, Zayed University, Abu Dhabi, United Arab Emirates; 4 Department of Computer Science, Al-Quds University, East Jerusalem, Occupied Palestinian Territory; 5 Department of Computer Engineering, Istinye University, Istanbul, Turkey; 6 Department of Clinical Nutrition and Dietetics, University of Sharjah, Sharjah, United Arab Emirates; 7 Nuffield Department of Women’s and Reproductive Health, University of Oxford, Oxford, United Kingdom; 8 Department of Nutrition and Health, College of Medicine and Health Sciences, United Arab Emirates University, Al Ain, United Arab Emirates; 9 Faculty of Public Health, Lebanese University, Beirut, Lebanon; 10 World Health Organization - Regional Office for the Eastern Mediterranean, Cairo, Egypt; 11 Department of Human Nutrition, College of Health Sciences, Qatar University, Doha, Qatar; 12 Department of Nutrition and Food Technology, Faculty of Agriculture, University of Jordan, Amman, Jordan; 13 National Nutrition Committee, Saudi Food and Drug Authority, Riyadh, Saudi Arabia; 14 Department of Clinical Nutrition, Faculty of Applied Medical Sciences, Taibah University, Medina, Saudi Arabia; 15 National Research Centre, Cairo, Egypt; 16 Public Authority for Applied Education and Training, Kuwait City, Kuwait; 17 Department of Biology, College of Science, University of Bahrain, Zallaq, Bahrain; 18 Food Science and Nutrition Department, College of Agricultural and Marine Sciences, Sultan Qaboos University, Muscat, Oman; 19 Faculty of Medicine, Al-Quds University, East Jerusalem, Occupied Palestinian Territory; 20 Doha Institute for Graduate Studies, Doha, Qatar; 21 Department of Nutrition and Food Technology, Faculty of Agriculture, Mu’tah University, Karak, Jordan; 22 Department of Mathematic, College of Science, University of Bahrain, Zallaq, Bahrain

**Keywords:** COVID-19, cigarette smoking, waterpipe smoking, body mass index, Arab countries

## Abstract

**INTRODUCTION:**

Tobacco smokers are at high risk of developing severe COVID-19. Lockdown was a chosen strategy to deal with the spread of infectious diseases; nonetheless, it influenced people’s eating and smoking behaviors. The main objective of this study is to determine the impact of the COVID-19 lockdown on smoking (waterpipe and cigarette) behavior and its associations with sociodemographic characteristics and body mass index.

**METHODS:**

The data were derived from a large-scale retrospective cross-sectional study using a validated online international survey from 38 countries (n=37207) conducted between 17 April and 25 June 2020. The Eastern Mediterranean Region (WHO-EMR countries) data related to 10 Arabic countries that participated in this survey have been selected for analysis in this study. A total of 12433 participants were included in the analysis of this study, reporting their smoking behavior and their BMI before and during the COVID-19 lockdown. Descriptive and regression analyses were conducted to examine the association between smoking practices and the participant’s country of origin, sociodemographic characteristics, and BMI (kg/m^2^).

**RESULTS:**

Overall, the prevalence rate of smoking decreased significantly during the lockdown from 29.8% to 23.5% (p<0.05). The percentage of females who smoke was higher than males among the studied population. The highest smoking prevalence was found in Lebanon (33.2%), and the lowest was in Oman (7.9%). In Egypt, Kuwait, Lebanon, and Saudi Arabia, the data showed a significant difference in the education level of smokers before and during the lockdown (p<0.05). Smokers in Lebanon had lower education levels than those in other countries, where the majority of smokers had a Bachelor’s degree. The findings show that the BMI rates in Jordan, Lebanon, Oman, and Saudi Arabia significantly increased during the lockdown (p<0.05). The highest percentages of obesity among smokers before the lockdown were in Oman (33.3%), followed by Bahrain (28.4%) and Qatar (26.4%), whereas, during the lockdown, the percentage of obese smokers was highest in Bahrain (32.1%) followed by Qatar (31.3%) and Oman (25%). According to the logistic regression model, the odds ratio of smoking increased during the pandemic, whereas the odds ratio of TV watching decreased. This finding was statistically significant by age, gender, education level, country of residence, and work status.

**CONCLUSIONS:**

Although the overall rates of smoking among the studied countries decreased during the lockdown period, we cannot attribute this change in smoking behavior to the lockdown. Smoking cessation services need to anticipate that unexpected disruptions, such as pandemic lockdowns, may be associated with changes in daily tobacco consumption. Public health authorities should promote the adoption of healthy lifestyles to reduce the long-term negative effects of the lockdown.

## INTRODUCTION

Pandemic-related disruptions to daily routines have impacted multiple behavioral measures and health conditions, particularly during the peak of the lockdown period^1^. Tobacco users are at higher risk of more severe COVID-19 outcomes^2^. As a result, the COVID-19 pandemic should be considered an urgent priority in tobacco control efforts to reduce tobacco consumption, make tobacco users aware of their high risks, and create campaigns to target tobacco cessation. However, it should be noted that other stressors resulting from the pandemic and related lockdown orders, such as increased anxiety, social isolation, and economic concerns, may have increased. As a result, these stressors may lead to an increase in the initiation of tobacco use by non-users and/or increased product use among current users^3^. As a result, it is essential to understand how pandemic-related lockdown measures have impacted tobacco-related behavior.

According to data from the World Health Organization (WHO), smoking causes the deaths of 8 million people per year, where 7 million deaths are caused by the direct use of tobacco, and the rest from passive smoking^4^. Moreover, there is the misconception that waterpipes are less harmful than cigarettes^5^. Waterpipe smokers are more likely to be exposed to CO (carbon monoxide) than cigarette smokers. Yet, cigarettes and waterpipes have the same end products that contain cancer-causing chemicals (carcinogens) and nicotine that leads to addiction^6^. One waterpipe session is equivalent to smoking 100 cigarettes^7^.

Besides, people who are passive smokers breathe in the smoke from burning cigarettes that smokers exhale. Most exposure to passive smoking occurs in homes, cars, and public spaces^8^. Therefore, passive smoking increases the risk of tobacco-related diseases among adults and children. One systemic review conducted among university students from several Arab countries found that smoking rates (cigarettes and waterpipe) were highest in Egypt, Kuwait, and Kingdom of Saudi Arabia, with higher smoking prevalence among males than females in Yemen, Bahrain, Tunisia, Egypt, Palestine, Syria, and Jordan, with significant differences between the countries^9^. Another study on smoking among young people in 16 Arab countries indicated that 10.6% of the participants were current waterpipe smokers, with the highest rate being in Lebanon (34.2%) and the lowest in Oman (9%). Overall, the study indicated that boys were more likely than girls to smoke waterpipes in Palestine, Lebanon, and Jordan (43.8%, 38.6%, and 25.7%, respectively)^10^.

Tobacco smokers are at high risk of severe COVID-19^2^ due to chronic lung diseases, weak immunity, cross-infection as the smokers might share devices between them as most of the waterpipes are smoked socially with others, and poor hygiene practices^11^. Waterpipe is often used with a shared mouthpiece smoking instrument between smokers, which increases the risk of viral transmission^12^. One systematic review study found that smokers were 1.4 times more likely (RR=1.4; 95% CI: 0.98–2.00) to have severe symptoms of COVID-19, in addition, close to 2.4 times more likely to be admitted to an ICU, might need mechanical ventilation or even die compared to non-smokers (RR=2.4; 95% CI: 1.43–4.04)^13^.

Several contributing factors among smokers that can increase the severity of COVID-19 include age, gender, level of education, nationality, and body health status^14^. It is suggested that certain factors such as income, employment, medical conditions, location, and access to healthcare have links to different races, ethnicity, and nationalities, and thus influence the COVID-19 severity^15^. One descriptive study has quantified the impacts of quarantine on behaviors related to weight gain and obesity. The average BMI of the participants was 27 kg/m^2^, and almost all of the participants reported staying home more often than before the pandemic. Gaining weight of 5–6 kg was reported by 22% of the sample^16^. Moreover, another cross-sectional study conducted during the COVID-19 quarantine involved 407 Lebanese participants in order to measure the behavioral features of eating and found that the BMI was higher during quarantine^17^. Another study conducted during the lockdown in the United Arab Emirates (UAE) concerning food intake and decreased physical activity, found that an increase in weight often lead to an increase in smoking frequency. Thus, all these factors may increase the vulnerability of COVID-19 and worsen the severity of the disease^18^.

In relation to the pandemic and smoking practices, a study conducted in Ohio during the early pandemic period measured the perceived risk of infection associated with indoor smoking and found that the desire to quit among smokers during the outbreak was related to the perceived risk of COVI-19 infection and diabetes^19^. While on the other hand, another national study conducted in the UK revealed that the psychosocial stress of the pandemic increased the prevalence of smoking and had more effect on former smokers^20^. In addition, a meta-study conducted in 2020 found a significant association between smoking and the progression of COVID-19 infection^21^.

Studying the impact of lockdown on various populations and age groups revealed that the lockdown had a negative effect on elderly peoples’ feelings of loneliness, isolation, and despair^22^. Therefore, our study aims to assess the impact of the COVID-19 lockdown on smoking practices (cigarettes and waterpipe) and to identify the associations with selected sociodemographic factors, working status, and BMI. As far as we know, there has not been a thorough study on the relationship between lockdown and smoking behaviors and BMI in Arab countries.

## METHODS

### Study design and settings

A retrospective observational study with a cross-sectional design using an online validated survey was launched originally in 38 different countries, and information collected from 37207 participants. The Eastern Mediterranean Region (WHO-EMR countries) data related to 10 Arab countries (United Arab Emirates, Lebanon, Bahrain, Egypt, Jordan, Kuwait, Oman, Qatar, Saudi Arabia, and Palestine) were selected for analysis in this study. The international study protocol was approved by the Ethics Committee for the Social Sciences and Humanities of the University of Antwerp (Ref no: SHW_20_46). The survey was kept open between 17 April and 25 June 2020. The details of the study methodology have been described elsewhere^23-25^. In addition to the international ethical approval and the approval from the Ethics Advisory Committee on Social and Human Sciences of the University of Antwerp, ethical approval was sought on 26 April 2020 from Zayed university Ethics Committee before data collection (Ref. No.: ZU20_098_F). Before moving on to the survey question, respondents had to read the following: 1) what the study is about (objective); 2) who can participate; 3) what are the rights and responsibilities; and 4) the contact details of the principal investigators. Finally, they were asked to provide informed consent and say whether they were aged ≥18 years, immediately after the survey’s welcome page (https://osf.io/r9n25/, accessed on 15 July 2022).

### Population and sampling

Participants included in this study were aged ≥18 years, of both genders, and residing in any participating Arab country; those aged <18 years were excluded from this study. Convenience snowball sampling was used to recruit participants, and advertisements for the survey were conducted using different social network platforms, in addition to the research team’s academic networks, university mailings, and stakeholders. First, the international research team created and shared multiple social media banners on sites such as Facebook, Twitter, Instagram, Snapchat, and LinkedIn in both private and public online groups. Furthermore, an international press release was distributed to the spokespersons of the research teams in each country, who were able to distribute it to their local press. In addition, the international news agency Reuters produced a video article about the Corona Cooking Survey, which was distributed to several international press organizations. The Corona Cooking Survey was mentioned in newspapers, on radio or on news websites in 24 different countries, so it was helpful to share the link to a global web page where participants could find the right survey link for their own country^23-25^.

### Measurements, instruments, and tools of data collection

The questionnaire was a self-administered, validated, and the online survey was conducted using the software Qualtrics and took approximately 20 minutes to complete. All countries used the same survey structure as a starting point. Extra questions were added at the end of the questionnaire, and some questions were adapted to better reflect the sociocultural background (e.g. alcohol use in Arab countries). An *ad hoc* questionnaire, created for the present study, took into account items from other questionnaires such as Health Behavior in School-aged Children (HBSC) (http://www.hbsc.org/methods/, accessed on 15 July 2022). Moreover, to ensure the questionnaire was valid and reliable, the survey questionnaire and the additional questions were reviewed and discussed by all principal researchers of the 10 Arab countries that participated in this study via several ZOOM meetings and WhatsApp groups created for this research project.

The complete questionnaire consisted of six main parts in addition to the additional questions specific to each country or region: 1) profiling questions; 2) lockdown and consequences; 3) general food behavior; 4) grocery shopping; 5) cooking and baking; and 6) eating behavior. The last part of the questionnaire used in the 10 Arab countries included additional questions about dieting, physical (in)activity, body image, smoking behaviors (cigarettes and waterpipes), and self-reported weight and height. The reported body mass index (BMI, kg/m^2^) was categorized according to the WHO criteria into: underweight (<18.5), normal (18.5–24.9), overweight (25–29.9) and obese (≥30)^26^.

The survey questions were available in native Arabic and English, extending choices for the respondents. A total of 12433 participants, from 10 Arab countries, who reported their smoking behavior before and during COVID-19 confinement were included in this analysis. Changes in smoking behaviors during the COVID-19 confinement were analyzed and compared according to selected sociodemographic factors (gender, age, education level, and working status) and BMI.

### Statistical analysis

A χ^2^ test was used to compare categorical variables/groups before and during the lockdown. A binary logistic regression analysis was conducted to assess the association between smoking before and during the COVID-19 pandemic and the associated independent variables, including age groups, physical activity, country, educational level, gender, work status, and watching TV. Odds ratios (ORs) and their 95% confidence intervals (CIs) were used as indicators of levels of association. A p<0.05 was considered statistically significant. Statistical analysis was conducted on IBM SPSS Statistics, Version 25 (IBM, Armonk, NY, USA)^27^.

## RESULTS

### Prevalence of smoking by country

Table 1, which compares smoking prevalence across the 10 Arab countries included in this study, shows that the overall smoking prevalence rate was 30% prior to lockdown and decreased to 24% during the lockdown. Before the lockdown, the highest prevalence was in Jordan, Lebanon, and Palestine (38%, 38%, and 33%, respectively), and the lowest was in Oman (10%). While during the lockdown, the highest prevalence of smoking was in Lebanon (33%), and the lowest was in Oman (8%). Before the lockdown, the prevalence rate of cigarettes smoked daily (more than once) was 28%, with Egypt having the highest rate (42%) and Jordan and Palestine having the lowest (26% and 26%, respectively). However, during the lockdown, there was an increase in the prevalence rate of cigarettes smoked daily (more than once) to 32%, with Egypt having the highest rate (56%). Before the lockdown, the prevalence of waterpipe smoking (more than once a week and more than once a day) was 46.4% across all countries. It increased during the lockdown to 54%, with Lebanon, Jordan, and Palestine having the highest prevalence. There was a statistically significant difference between the countries (Lebanon, Palestine, and Jordan) and smoking (p<0.001), (Table 1).

**Table 1:** Smoking practices (cigarettes and waterpipes) before and during COVID-19 lockdown among participants of 10 Arab countries, 2020 (N=12433)

*Smoking practices*	*Bahrain*		*Egypt*		*Jordan*		*Kuwait*		*Lebanon*		*Oman*		*Qatar*		*Saudi Arabia*		*Emirates*		*Palestine*		*Total*	*p*
	*n*	*%*	*n*	*%*	*n*	*%*	*n*	*%*	*n*	*%*	*n*	*%*	*n*	*%*	*n*	*%*	*n*	*%*	*n*	*%*	*n*	
**Smoking before**																						
No	534	82.8	480	71.6	1578	62.2	451	69.4	1321	62.5	148	90.2	434	75.6	2101	76.7	1223	79.4	537	67.5	9729	0.000
Yes	111	17.2	190	28.4	961	37.8	199	30.6	794	37.5	16	9.8	140	24.4	640	23.3	317	20.6	258	32.5	4102	
**Smoking during**																						
No	563	87.3	538	80.3	1741	68.6	515	79.2	1412	66.8	151	92.1	472	82.2	2303	84.0	1313	85.3	567	71.3	10580	0.000
Yes	82	12.7	132	19.7	798	31.4	135	20.8	703	33.2	13	7.9	102	17.8	438	16.0	227	14.7	228	28.7	3251	
**Smoking cigarettes before**																						
Almost never	64	57.7	76	40.0	570	59.3	102	51.3	467	58.8	5	31.3	72	51.4	376	58.8	164	51.7	164	63.6	2349	0.000
> once a week	11	9.9	35	18.4	146	15.2	27	13.6	106	13.4	3	18.8	20	14.3	74	11.6	59	18.6	28	10.9	598	
≥ once a day	36	32.4	79	41.6	245	25.5	70	35.2	221	27.8	8	50.0	48	34.3	190	29.7	94	29.7	66	25.6	1155	
**Smoking cigarettes during**																						
Almost never	38	46.3	30	22.7	405	50.8	48	35.6	375	53.3	3	23.1	45	44.1	213	48.6	90	39.6	129	56.6	1586	0.000
> once a week	17	20.7	28	21.2	156	19.5	21	15.6	107	15.2	3	23.1	20	19.6	64	14.6	58	25.6	30	13.2	609	
≥ once a day	27	32.9	74	56.1	237	29.7	66	48.9	221	31.4	7	53.8	37	36.3	161	36.8	79	34.8	69	30.3	1056	
**Smoking waterpipes before**																						
Almost never	63	56.8	148	77.9	468	48.7	137	68.8	345	43.5	7	43.8	98	70.0	376	58.8	193	60.9	137	53.1	2214	0.000
> once a week	30	27.0	29	15.3	311	32.4	31	15.6	227	28.6	6	37.5	34	24.3	155	24.2	76	24.0	70	27.1	1106	
≥ once a day	18	16.2	13	6.8	182	18.9	31	15.6	222	28.0	3	18.8	8	5.7	109	17.0	48	15.1	51	19.8	782	
**Smoking waterpipes during**																						
Almost never	43	52.4	109	82.6	328	41.1	84	62.2	270	38.4	4	30.8	62	60.8	213	48.6	116	51.1	107	46.9	1494	0.000
> once a week	24	29.3	14	10.6	284	35.6	23	17.0	192	27.3	5	38.5	28	27.5	107	24.4	63	27.8	65	28.5	949	
≥ once a day	15	18.3	9	6.8	186	23.3	28	20.7	241	34.3	4	30.8	12	11.8	118	26.9	48	21.1	56	24.6	808	

p<0.001.

### Prevalence of smoking by gender

**Figure 1:**
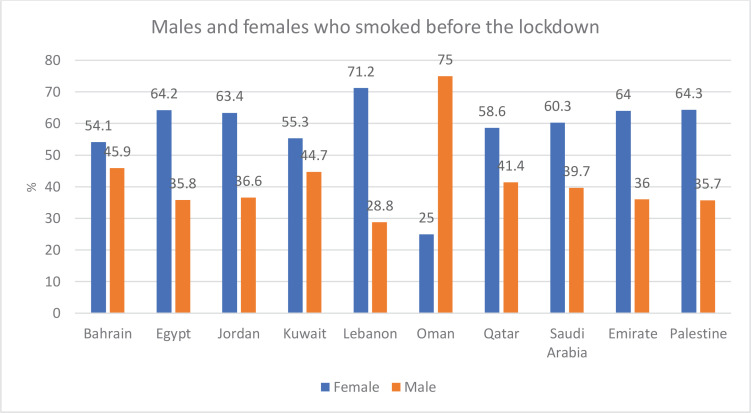
The percentage of males and females who smoked before the lockdown in 10 Arab countries

According to the data, the prevalence of female smokers was highest in Lebanon (71%), followed by Palestine (64%), Egypt (64%), the United Arab Emirates (64%), Jordan (63%), Saudi Arabia (60%), Qatar (58.6%), Kuwait (55%), and Bahrain (54%), and the lowest was in Oman (25%). On the other hand, the percentage of males who smoked before the lockdown was higher in Oman (75%) only (Figure 1).

**Figure 2:**
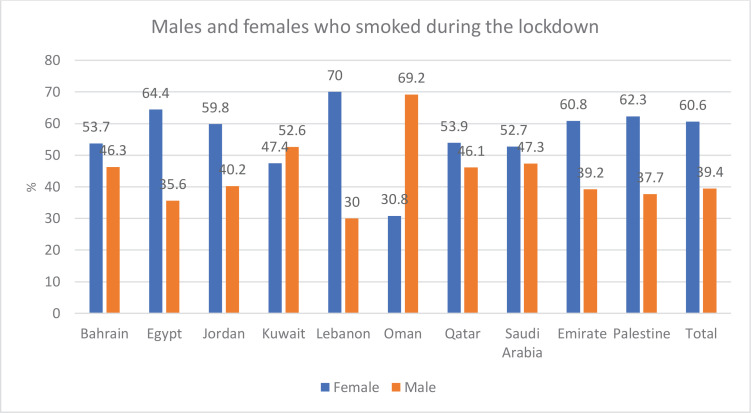
The percentage of males and females who smoked during the lockdown in 10 Arab countries

During the lockdown, Lebanon had the highest percentage of female smokers (70%) followed by Egypt (64%), Palestine (62%), the United Arab Emirates (61%), Jordan (60%), Qatar (54%), Bahrain (54%), and Saudi Arabia (53%). In contrast, Kuwait (53%) and Oman (69%) had a higher proportion of men than women who smoked during the lockdown (Figure 2).

By comparing the percentages of female smokers before and during the lockdown in the 10 Arab countries, it was found that, except for Egypt and Oman, the percentage of female smokers before the lockdown was higher than the percentage of female smokers during the lockdown. In Egypt, the percentage of women who smoked before the lockdown was 64.2% and slightly increased during the lockdown to 64.4%. In Oman, the rate of females who smoke increased from 25% before the lockdown to 31% during the lockdown. In contrast, the data show that, with the exception of Egypt and Oman where the percentage of males who smoke was higher before the lockdown and decreased during the lockdown, the percentage of males who smoke before the lockdown increased during the lockdown (Figures 1 and 2). Results across the 10 Arab countries showed that males had a significant difference from female, before and during the lockdown (p<0.05).

### Prevalence of smoking by age

Table 2 provides information on the various age categories of smokers in the 10 Arab countries before and during the lockdown. In Bahrain, Jordan, Lebanon, Oman, Qatar, Saudi Arabia, the United Arab Emirates, and Palestine, most smokers are between the ages of 20 and 29 years. In Kuwait, most smokers are aged between 40 and 49 years. In Egypt, the majority of smokers are 50 years of age or older.

**Table 2:** Sociodemographic characteristics, work status, and BMI, before and during COVID-19 among participants of 10 Arab countries, 2020 (N=12433)

*Characteristics*	*Bahrain*		*Egypt*		*Jordan*		*Kuwait*		*Lebanon*		*Oman*		*Qatar*		*Saudi Arabia*		*Emirates*		*Palestine*		*Total*	*p*
	*n*	*%*	*n*	*%*	*n*	*%*	*n*	*%*	*n*	*%*	*n*	*%*	*n*	*%*	*n*	*%*	*n*	*%*	*n*	*%*	*n*	
**Gender**																						
Female	525	81.4	510	76.1	1982	78.1	507	78.0	1706	80.7	138	84.1	454	79.1	2241	81.8	1253	81.4	635	79.9	9951	0.006
Male	120	18.6	160	23.9	557	21.9	143	22.0	409	19.3	26	15.9	120	20.9	500	18.2	287	18.6	160	20.1	2482	
**Age** (years)																						
18–19	37	5.7	25	3.7	355	14.0	48	7.4	372	17.6	5	3.0	58	10.1	195	7.1	159	10.3	58	7.3	1312	0.000
20–29	257	39.8	152	22.7	1664	65.5	168	25.8	1004	47.5	59	36.0	275	47.9	1343	49.0	890	57.8	362	45.5	6174	
30–39	150	23.3	117	17.5	245	9.6	165	25.4	326	15.4	49	29.9	96	16.7	718	26.2	229	14.9	157	19.7	2252	
40–49	104	16.1	165	24.6	179	7.1	147	22.6	227	10.7	39	23.8	92	16.0	336	12.3	159	10.3	149	18.7	1597	
≥50	97	15.0	211	31.5	96	3.8	122	18.8	186	8.8	12	7.3	53	9.2	149	5.4	103	6.7	69	8.7	1098	
**Education level**																						
≤ High school diploma	178	27.6	76	11.3	515	20.3	175	26.9	742	35.1	27	16.5	155	27.0	760	27.7	474	30.8	92	11.6	3194	0.000
Bachelor’s degree	381	59.1	376	56.1	1897	74.7	369	56.8	848	40.1	87	53.0	306	53.3	1712	62.5	846	54.9	539	67.8	7361	
Graduate degree	86	13.3	218	32.5	127	5.0	106	16.3	525	24.8	50	30.5	113	19.7	269	9.8	220	14.3	164	20.6	1878	
**Work before**																						
Unemployed	340	52.7	243	36.3	1936	76.3	269	41.4	1258	59.5	59	36.0	311	54.2	1745	63.7	983	63.8	392	49.3	7536	0.000
Employed	305	47.3	427	63.7	603	23.7	381	58.6	857	40.5	105	64.0	263	45.8	996	36.3	557	36.2	403	50.7	4897	
**Work during**																						
Unemployed	369	57.2	323	48.2	2079	81.9	462	71.1	1450	68.6	77	47.0	331	57.7	2009	73.3	1010	65.6	485	61.0	8595	0.000
Employed	276	42.8	347	51.8	460	18.1	188	28.9	665	31.4	87	53.0	243	42.3	732	26.7	530	34.4	310	39.0	3838	
**BMI before**																						
Underweight	42	6.9	7	1.1	204	8.4	17	2.8	161	7.9	17	10.8	29	5.4	227	8.7	120	8.2	35	4.6	859	0.000
Normal	233	38.2	222	34.5	1346	55.1	235	38.0	1135	55.6	63	40.1	202	37.4	1092	42.0	698	47.8	384	50.7	5610	
Overweight	171	28.0	238	37.0	579	23.7	183	29.6	482	23.6	39	24.8	173	32.0	702	27.0	355	24.3	223	29.5	3145	
Obese	164	26.9	176	27.4	312	12.8	183	29.6	264	12.9	38	24.2	136	25.2	579	22.3	286	19.6	115	15.2	2253	
**BMI during**																						
Underweight	48	8.0	8	1.3	203	8.4	21	3.5	145	7.2	17	11.1	29	5.4	219	8.5	119	8.2	29	3.8	838	0.000
Normal	211	35.0	184	28.8	1271	52.7	209	34.7	1085	54.1	61	39.9	204	38.0	1103	42.7	664	45.9	377	49.4	5369	
Overweight	172	28.5	252	39.4	605	25.1	200	33.2	519	25.9	40	26.1	181	33.7	701	27.1	388	26.8	229	30.0	3287	
Obese	172	28.5	195	30.5	335	13.9	173	28.7	256	12.8	35	22.9	123	22.9	560	21.7	275	19.0	128	16.8	2252	

BMI: body mass index (kg/m^2^). p<0.006.

Overall, the percentage of smokers aged between 20 and 29 years decreased during the lockdown in Bahrain, Qatar, Saudi Arabia, and the United Arab Emirates. Similarly, in Egypt, the number of smokers aged ≥50 years decreased during the lockdown. On the other hand, the percentage of smokers aged between 20 and 29 years increased in Jordan, Lebanon, and Oman during the lockdown. Also, in Kuwait, the number of smokers aged ≥50 years increased during the lockdown.

In conclusion, smokers in Jordan, Lebanon, Oman, and Kuwait increased their smoking during the lockdown. In contrast, most smokers in Bahrain, Qatar, Saudi Arabia, the United Arab Emirates, and Egypt decreased their smoking. In Palestine, smokers aged 20–29 years show a consistent percentage before and during the lockdown. In Egypt, Jordan, Lebanon, Qatar, Saudi Arabia, and the United Arab Emirates, data showed that the second and third age groups (20–29; 30–39 years) had significant difference at p<0.05.

### Prevalence of smoking by education level

According to Table 2, most participants who smoked before and during the lockdown in Bahrain, Egypt, Jordan, Kuwait, Oman, Qatar, Saudi Arabia, the United Arab Emirates, and Palestine had Bachelor’s degrees. In Lebanon, most smokers had a high school diploma or less. The findings indicate that, during the COVID-19 pandemic, smoking rates among those with a Bachelor’s degree increased in Egypt, Oman, Saudi Arabia, and Palestine while decreased in Bahrain, Jordan, Kuwait, and the United Arab Emirates. In Lebanon, smoking increased during the lockdown. In Egypt, Kuwait, Lebanon, and Saudi Arabia, the data showed a significant difference in the education level of smokers before and during the lockdown (p<0.05).

### Prevalence of smoking by working status

Before the lockdown, the prevalence of unemployment among the overall sample was 60%. This percentage increased to 69% during the lockdown. Countries that showed the highest increase in the unemployment rate were Jordan (82%), Kuwait (72%), Lebanon (69%), and Palestine (61%) (Table 2).

### Prevalence of smoking by BMI

Before the lockdown, Oman (33%), Bahrain (28%), and Qatar (26%) had the highest rates of obesity among smokers. During the lockdown, Bahrain (32%), Qatar (31%), and Oman (25%) had the highest rates of obesity among smokers. As observed in the data results, the percentage of obesity among smokers increased during the lockdown in both Bahrain and Qatar, while it decreased in Oman. Before the lockdown, the percentage of overweight smokers was highest in Egypt (37%) followed by Qatar (36%) and Oman (33%).

In addition, during the lockdown, the percentage of smokers who were overweight was highest in Egypt (43%), followed by the United Arab Emirates (39%) and Qatar (38%). According to the data, while the percentage of overweight among smokers in Egypt and Qatar increased slightly during the lockdown, the overweight among smokers in the United Arab Emirates increased significantly. Before the lockdown, the percentage of overweight among smokers in the United Arab Emirates was 28%; during the lockdown, it increased to 39%, indicating a significant increase in weight among smokers. The data reveal a considerable change in BMI between before and during the lockdown in Jordan, Lebanon, Oman, and Saudi Arabia, the overweight and obese participants had significant differences from normal BMI participants.

### Binary logistic regression analysis

Logistic regression was performed to ascertain the effects of the COVID-19 pandemic on smoking by age, gender, country of residence, physical activity levels, and participants working status. Table 3 shows the binary logistic regression analysis of smoking by sociodemographic variables. The logistic regression model was statistically significant by age, gender, education level, country of residence, work status, and watching TV activity. The regression model revealed an increase in smoking by age (20–29 and 30–39 years) during the COVID-19 lockdown compared to smoking before; the estimated odds ratio between smoking before and during the lockdown for the age groups 20–29 and 30–39 years, ranged 1.29–1.41 and 1.39–1.52, respectively. Furthermore, the results show increases in the odds ratio between smoking before and during the COVID-19 lockdown by gender, country of residence (Jordan, Lebanon, and Palestine), and working status. An interesting finding was a decrease in the smoking rate during the COVID-19 pandemic compared with smoking before the pandemic, according to TV watching activity.

**Table 3:** Binary logistic regression analysis of smoking before and during COVID-19 lockdown by age, physical activity, country, education level, gender, work status, and watching TV, 2020 (N=12433)

*Characteristics*	*Smoking before COVID-19*		*Smoking during COVID-19*	
	*OR (95% CI)*	*p*	*OR (95% CI)*	*p*
18–19 (Ref.)	1		1	
20–29	1.29 (1.1–1.5)	0.001	1.39 (1.2–1.6)	0.001
30–39	1.41 (1.2–1.7)	0.001	1.52 (1.3–1.9)	0.001
40–49	1.13 (0.9–1.4)	0.213	1.12 (0.9–1.4)	0.297
≥50	0.86 (0.7–1.1)	0.178	0.89 (0.7–1.1)	0.293
High (Ref.)	1		1	
Inactive	0.97 (0.9–1.1)	0.601	1.01 (0.9–1.2)	0.878
Low	0.99 (0.9–1.1)	0.859	0.95 (0.8–1.1)	0.544
Moderate	0.97 (0.9–1.1)	0.606	1.04 (0.9–1.2)	0.589
Other (Ref.)	1		1	
Bahrain	0.36 (0.3–0.5)	0.001	0.33 (0.2–0.4)	0.001
Egypt	0.72 (0.6–0.9)	0.003	0.56 (0.4–0.7)	0.001
Jordan	1.21 (1.1–1.4)	0.009	1.22 (1.0–1.4)	0.012
Kuwait	0.78 (0.6–1)	0.02	0.59 (0.5–0.7)	0.001
Lebanon	1.2 (1.0–1.4)	0.014	1.32 (1.1–1.5)	0.001
Oman	0.2 (0.1–0.3)	0.001	0.21 (0.1–0.4)	0.001
Qatar	0.59 (0.5–0.7)	0.001	0.51 (0.4–0.7)	0.001
Saudi Arabia	0.55 (0.5–0.6)	0.001	0.44 (0.4–0.5)	0.001
Emirate	0.48 (0.4–0.6)	0.001	0.4 (0.3–0.5)	0.001
Palestine	0.93 (0.8–1.1)	0.458	1.03 (0.8–1.3)	0.763
Graduate degree (Ref.)	1		1	
Lower or high school diploma	1.43 (1.2–1.6)	0.001	1.58 (1.4–1.8)	0.001
Bachelor’s degree	1.2 (1.1–1.3)	0.002	1.28 (1.1–1.5)	0.001
Male (Ref.)	1		1	
Female	3.52 (3.2–3.9)	0.001	3.79 (3.4–4.2)	0.001
Student (Ref.)	1		1	
Employed	1.43 (1.3–1.6)	0.001	1.68 (1.5–1.9)	0.001
Unemployed	1.16 (1.0–1.3)	0.02	1.39 (1.2–1.6)	0.001
<1 (Ref.)	1		1	
1–2	1.08 (1–1.2)	0.116	0.7 (0.6–0.8)	0.001
3–4	1.27 (1.1–1.4)	0.001	0.81 (0.7–0.9)	0.001
≥5	1.4 (1.2–1.7)	0.001	0.8 (0.7–0.9)	0.001

p<0.05.

## DISCUSSION

This cross-sectional study offers information about the effects of COVID-19 on cigarette and waterpipe smoking among people by gender, age, educational level, and BMI, in the 10 Arab countries that participated in an online survey following the lockdown to stop the spread of COVID-19 and lower the prevalence of smoking among people living in these countries.

According to our data, smoking had generally decreased during the lockdown in most Arab countries. However, it remained high in Lebanon, Jordan, and Palestine; this might be related to the economic stress experienced in these low-income countries where people were either unemployed or, at most, receiving half their typical salary for more than four months; the lockdown posed significant difficulties and limitations. This finding was similar to that of a study conducted in the middle African region^21^. Smoking was one of the psychological responses used by young adults to deal with the stress and anxiety associated with meeting their basic needs. One study found that smoking was significantly associated with stress and psychological distress among adolescent refugees in Lebanon, Jordan, Palestine, and Syria^28^.

Additionally, a study conducted by Chezhian et al.^29^ revealed that stress was one of the contributing factors that led people to smoke in general situations. Unemployment was found in our study to increase smoking during the lockdown and to be associated with smoking^30^. Furthermore, based on our study results, Lebanon shows a high percentage of smoking among females and males before and during the lockdown. According to Gulf News, Lebanon has ranked third in the world for the highest tobacco consumption per capita and that smoking is considered one of the cultural practices in Lebanon^31^. Additionally, a study reveals that due to Lebanon’s extensive tobacco growing, the availability and access to tobacco is easier and the average monthly consumption of cigarette packs is 12.4 packs (2008–2009 study), which exceeds that in Jordan (3.7 packs) and Syria (4.4 packs)^32^. During the pandemic, people also experience higher fear, higher boredom, and higher anxiety from the COVID-19 disease spread and lockdown, as found by Haddad et al.^17^ in a study in Lebanon.

Interestingly, in most of the 10 Arab countries, except for Oman, Qatar, and Kuwait, the smoking rate among women was greater than that of men both before and during the lockdown, with a higher odds ratio during the pandemic lockdown. This result was inconsistent with a study by Kashyap et al.^33^, and might be due to the higher percentage of women who participated in their study. Other studies show that men smoke more often than women, which increases the susceptibility and severity of COVID-19 among men rather than women^34^. However, our study’s findings may be explained by the fact that Arab women were not adapting to the presence of men at home all the time, as well as by the transition of children to online education, which added more pressure and stress on women. In addition, based on a UN women’s organization^35^, the reported cases of violence against Arab women increased during the lockdown; which showed that, after considering all the economic, social, and cultural views of Arab countries, the reported violence (physical, psychological, and sexual) during the lockdown was significantly higher than previously. Due to the restrictions on movement during the lockdown, women tend not to ask for assistance and receive little help from even their extended families. Given the challenging circumstances they endure and the fact that many women are forced to live with their abusers in the same house day and night, smoking may be the only coping mechanism they have access to. Although smoking was linked to violence in earlier research before the pandemic, our study may be the first to connect smoking with gender during the lockdown. The earlier studies found that women who experience violence increase their smoking behavior^36,37^.

Education was one of the most important results that our study showed. Despite having a higher level of education (at the university Bachelor’s level), smoking rates among students increased during the lockdown in most studied countries. Formal education level was positively associated with the cessation of smoking and not smoking in previous studies^38^.

Age was another factor we examined in our study, and we found that younger ages, between 20 and 29 years, may be associated with economic stressors or free time during the lockdown. According to our research, Egypt had the highest prevalence of smokers among those aged ≥50 years, with a rate of 31.8%. This result was similar to a cross-sectional study conducted in Egypt where the prevalence rate was 25.3%^39^. Therefore, increasing the percentage of active smokers among the elderly might increase the Case Fatality Rate in Egypt, as mentioned in a study that COVID-19 attacks the elderly more aggressively than children^40^. Furthermore, tobacco smokers are at high risk of severe COVID-19 due to chronic lung diseases, weak immunity, and cross-infection, as the smokers share devices between them with poor hygiene practices.

Regarding obesity and overweight among the smokers before and during the lockdown, the highest percentage of obese smokers before the lockdown was in Oman (33%), followed by Bahrain (28%) and Qatar (26%), whereas, during the lockdown, the percentage of obese smokers was highest in Bahrain (32%) followed by Qatar (31.3%) and Oman (25%). Results revealed that most Gulf Cooperation Council countries have high rates of obesity among both genders, which may increase the burden of disease in the future. In a study by Radwan et al.^18^, the lockdown in the UAE resulted in higher food consumption and lower levels of physical activity. Therefore, an increase in weight is experienced with an increase in smoking frequency. These factors can increase vulnerability of being infected by COVID-19 and worsen the severity of the disease, which was consistent with our study. The percentage of overweight smokers in the UAE before the lockdown was 28%, whereas during the lockdown it increased to 39%. Obesity and overweight did not increase in low-income countries during the lockdown since food consumption did not rise, and in some cases, people suffered from a lack of resources.

Interestingly, our study shows a significant association between watching TV and a decrease in smoking behavior during the lockdown, as the more hours spent watching TV, the lower the odds ratio with smoking during the lockdown. During the lockdown, people face fear, loneliness, and eagerness to follow up on the pandemic distribution and lockdown updates; watching TV was one of the strategies to deal with all those feelings found in other studies^19,20^. In addition, to the fact that people understand the effect of second-hand smoking on infectious diseases such as COVI-19, smoking while watching TV is reduced, and people tend to relapse from smoking while watching TV. Since they spend more time at home watching TV, simultaneously the smoking rate will be lower^19^.

### Strengths and limitations

When interpreting the results, it is necessary to consider the study’s strengths and limitations. The diversity of the sample from 10 Arab countries is a study strength. This study is the first, that we are aware of, that attempts to quantify changes in smoking practices in numerous Arab countries. The results of this study may thus be applied to the entire population of the 10 Arab countries during the lockdown. Besides the strengths of this study, it is not without limitations. One of the limitations is related to the data collection before and during COVID-19 based on self-report, which might have resulted in participant recall bias. Another limitation is associated with the comparison of the current study with other similar studies by using different methods to measure smoking and assess screen time, making direct comparisons with results and conclusions difficult. Using a cross-sectional study design limits ascertaining causality relationships. Moreover, the study’s convenience sampling might limit generalizability. Cross-cultural differences between countries are considered another limitation for interpretations and comparisons.

## CONCLUSIONS

Internationally, many strategies have been advocated to prevent the spread of COVID-19, including social distancing, strict hygiene, and extreme circumstances like lockdown. A lockdown was implemented during COVID-19 as a quick action to limit the spread of the disease. However, this action had many unintended consequences, some of which were obvious, like economic issues, and others not so obvious, like smoking, female smoking rates rising, and increased food consumption and obesity.

Special attention must be paid to illness prevention and health promotion, with a focus on smoking and diseases like COVID-19 and other respiratory conditions. During the lockdown, or other infection control in the event of a pandemic, a healthy lifestyle must be supported and promoted. Better preparedness and planning for health promotion campaigns must accompany any lockdown strategies to combat infectious diseases in the future, including reducing smoking and encouraging a healthy lifestyle.

## Data Availability

The data supporting this research are available from the authors on reasonable request.

## Notes

*Regional CORONA COOKING Survey Group:

**Belgium:** Charlotte De Backer, Kathleen Van Royen, Lauranna Teunissen, Isabelle Cuykx, Paulien Decorte, Gaëlle Ouvrein, Karolien Poels, Heidi Vandebosch, Katrien Maldoy (University of Antwerp); Sara Pabian (Tilburg University), Christophe Matthys, Tim Smits, Jules Vrinten (KULeuven); Ann DeSmet (University of Antwerp, Université Libre de Bruxelles); Nelleke Teughels, Maggie Geuens, Iris Vermeir, Viktor Proesmans, Liselot Hudders (Ghent University)

**Bahrein:** Tariq Abdulkarim Alalwan

**Jordan:** Nahla Al-Bayyari (Al-Balqa Applied University), Mohammed O. Ibrahim (Mu’tah University), Fadwa Hammouh (American University of Madaba)

**Kuwait:** Basma Dashti (Kuwait Institute for Scientific Research), Dhuha Alkharaif (Public Authority for Applied Education & Training), Amani Alshatti (Public Authority for Applied Education & Training), Maryam Al Mazedi (Public Authority for Applied Education & Training)

**Lebanon:** Elissa Naim, Hussein Mortada (Lebanese University), Carla Ibrahim (Lebanese University & Holy Spirit University of Kaslik)

**Palestine:** Motasem Hamdan, Hazem Agha (Al Quds University)

**Qatar:** Manal Othman (Hamad Medical Corporation), Jaafar Pakari, Rasha Abu-El- Ruz (Qatar University; QU-Health)

**Saudi Arabia:** Jamila Arrish, Ghadir A. Fallata, Omar A. Alhumaidan, Shihana A. Alakeel, Norah A. AlBuayjan, Sarah M. Alkhunein, Budur M. Binobaydan, Aeshah A. Alshaya [National Nutrition Committee (NNC) at Saudi Food and Drug Authority (Saudi FDA)]

**United Arab Emirates:** Sharifa AlBloosh
